# Dynamic Content Reactivation Supports Naturalistic Autobiographical Recall in Humans

**DOI:** 10.1523/JNEUROSCI.1490-20.2020

**Published:** 2021-01-06

**Authors:** Adrian W. Gilmore, Alina Quach, Sarah E. Kalinowski, Stephen J. Gotts, Daniel L. Schacter, Alex Martin

**Affiliations:** ^1^Section on Cognitive Neuropsychology, Laboratory of Brain and Cognition, National Institute of Mental Health, National Institutes of Health, Bethesda, Maryland 20892; ^2^Department of Psychology, Harvard University, Cambridge, Massachusetts 02138

**Keywords:** autobiographical interview, autobiographical memory, fMRI, reactivation, spoken recall

## Abstract

Humans can vividly recall and re-experience events from their past, and these are commonly referred to as episodic or autobiographical memories. fMRI experiments reliably associate autobiographical event recall with activity in a network of “default” or “core” brain regions. However, as prior studies have relied on covert (silent) recall procedures, current understanding may be hampered by methodological limitations that obscure dynamic effects supporting moment-to-moment content retrieval. Here, fMRI participants (*N* = 40) overtly (verbally) recalled memories for ∼2 min periods. The content of spoken descriptions was categorized using a variant of the Autobiographical Interview (AI) procedure (Levine et al., 2002) and temporally re-aligned with BOLD data so activity accompanying the recall of different details could be measured. Replicating prior work, sustained effects associated with autobiographical recall periods (which are insensitive to the moment-to-moment content of retrieval) fell primarily within canonical default network regions. Spoken descriptions were rich in episodic details, frequently focusing on physical entities, their ongoing activities, and their appearances. Critically, neural activity associated with recalling specific details (e.g., those related to people or places) was transient, broadly distributed, and grounded in category-selective cortex (e.g., regions related to social cognition or scene processing). Thus, although a single network may generally support the process of vivid event reconstruction, the structures required to provide detail-related information shift in a predictable manner that respects domain-level representations across the cortex.

**SIGNIFICANCE STATEMENT** Humans can vividly recall memories of autobiographical episodes, a process thought to involve the reconstruction of numerous distinct event details. Yet how the brain represents a complex episode as it unfolds over time remains unclear and appears inconsistent across experimental traditions. One hurdle is the use of covert (silent) in-scanner recall to study autobiographical memory, which prevents experimenter knowledge of what information is being retrieved, and when, throughout the remembering process. In this experiment, participants overtly described autobiographical memories while undergoing fMRI. Activity associated with the recall and description of specific details was transient, broadly distributed, and grounded in category-selective cortex. Thus, it appears that as events unfold mentally, structures are dynamically reactivated to support vivid recollection.

## Introduction

Humans have a potentially unique capacity to vividly re-experience events from their past (Tulving, 1983, 1985). The constructive episodic simulation (CES) hypothesis (Schacter and Addis, 2007) suggests that mental time travel is enabled by reconstructive aspects of human memory (see also Bartlett, 1932; Schacter et al., 1998; Hassabis and Maguire, 2007). The hypothesis states that during an initial “construction” phase, different elements (“details”) of an experience are recombined into coherent event representation via hippocampally-mediated processes, and the event subsequently plays out in one's mind during an “elaboration” phase (Addis et al., 2007; Addis and Schacter, 2012). The mechanisms supporting elaboration are less clearly specified, yet it is this aspect of recall that seems to give mental time travel its unique phenomenology (Tulving, 2002). One must therefore ask: how are details experienced in “real time” as we remember?

One possibility is that as each detail is recalled, the brain draws on a distributed collection of regions that support knowledge of that domain or category (Martin, 2007, 2016). Such “reactivation” effects are also predicted by computational models of episodic memory (Norman and O'Reilly, 2003); by fMRI studies that directly manipulate encoding modalities (Wheeler et al., 2000), study categorized list recall (Polyn et al., 2005), or seek to decode information from visual memory (Bone et al., 2020); and by studies using intracranial electrodes that observe “ripples” traveling from category-selective ventral temporal regions to medial temporal lobe structures (Norman et al., 2019; Vaz et al., 2019). Thus, one might expect to observe dynamic reactivation effects throughout an episode's recall. However, fMRI studies of autobiographical retrieval paint a decidedly different picture. These have, instead, found evidence for a single network supporting episodic autobiographical recall (Svoboda et al., 2006; McDermott et al., 2009; Boccia et al., 2019; Ritchey and Cooper, 2020). Whether they are referred to as members of the default network (Andrews-Hanna, 2012), autobiographical memory network (Svoboda et al., 2006), posterior medial system (Ranganath and Ritchey, 2012), or simply the core network (Benoit and Schacter, 2015), the same basic regions are reliably activated when remembering events from one's past.

Several explanations for the discrepancy exist. Perhaps, during the initial construction phase, all the details associated with an event are activated and maintained in default/core network regions (for related discussions, see Benoit et al., 2014; Szpunar et al., 2014; Thakral et al., 2017, 2020; see also Ritchey and Cooper, 2020). In this view, detail reactivation during the elaboration phase would be redundant. Alternatively, it may be that typical autobiographical memory experiments are structured systematically in ways that paint an incomplete picture of event retrieval. In particular, the use of covert (silent) recall in fMRI studies reduces participant head motion but prohibits experimental knowledge of retrieval dynamics: of what is recalled, and when. Consequently, regions associated with the (sustained) online manipulation of retrieved details might be observed, but those involved with (transient) detail reactivation might not.

**Figure 1.**
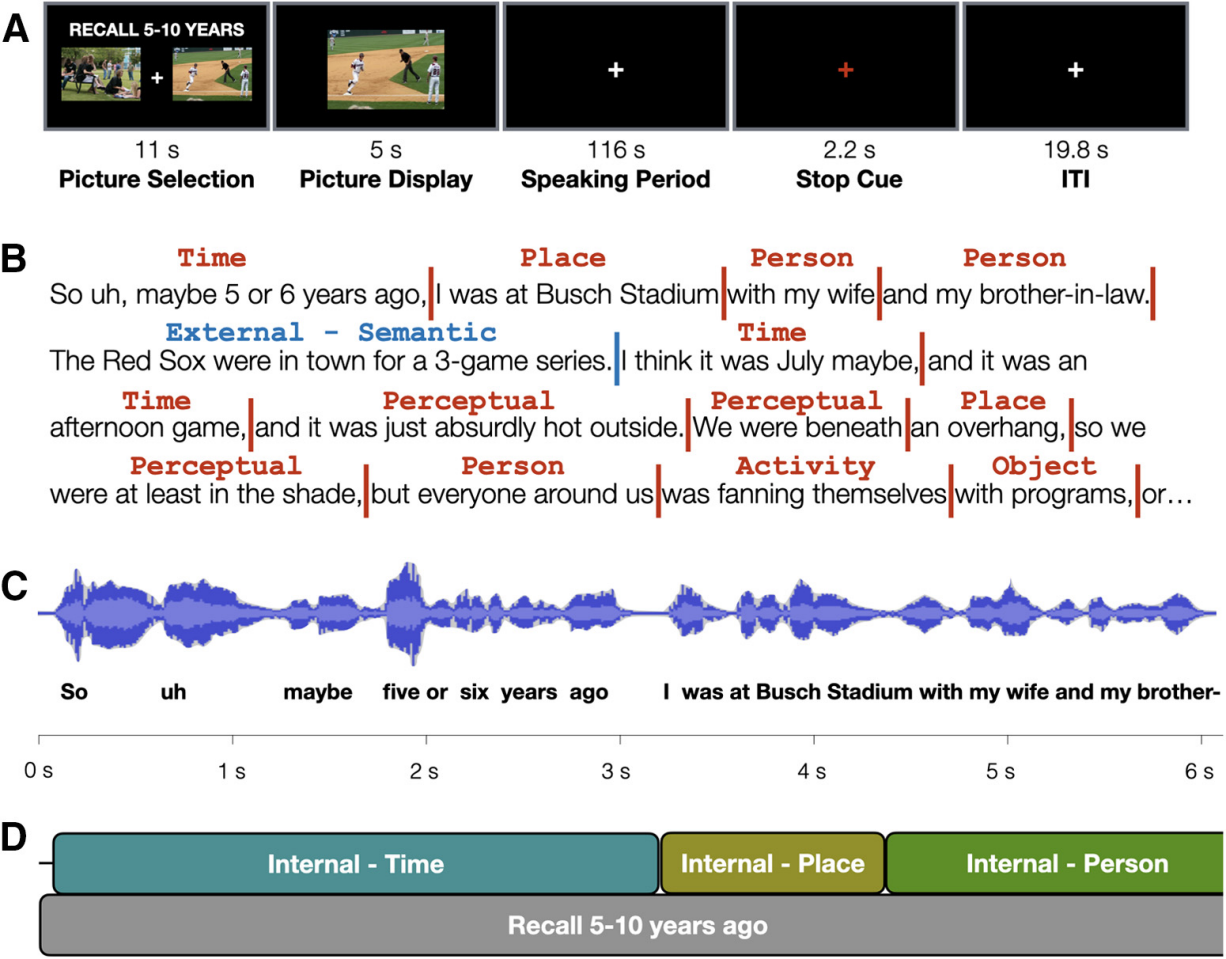
Approach and design. ***A***, Participants were cued to overtly recall specific memories from their past or describe the contents of a complex photograph (condition not shown). ***B***, Transcripts of each event description were scored for the type and content of each detail using an adapted form of the AI (Levine et al., 2002). ***C***, Each transcribed word was temporally aligned with the original audio to determine the onset time and duration of each scored event detail. Words are spaced to reflect the relative temporal separation derived from the audio alignment. ***D***, This information was used to create regressors for use with fMRI timeseries data, enabling an event-related analysis of the naturalistically recalled content.

Here, we used overt, within-scanner naturalistic recall to study episodic autobiographical retrieval. Participants (*N* = 40) verbally recalled autobiographical events in response to photographic picture cues, and for each recalled memory were given ∼2 min to describe the event as it unfolded in their mind (Fig. 1*A*). A non-autobiographical control task required participants to describe the complex photographic cues (see Gaesser et al., 2011; Madore et al., 2014) immediately after viewing rather than using them to retrieve past episodes. Transcribed verbal reports of each memory were broken down into different content types using an adapted version of the Autobiographical Interview (AI) scoring procedure (Levine et al., 2002; Fig. 1*B*), which is commonly used in behavioral studies to identify differences in recalled content (Addis et al., 2009b; Irish et al., 2018). Text was time stamped based on the original audio and synchronized with the BOLD timeseries (Fig. 1*C*). In this way, recalled details could be leveraged to study the dynamic nature of ongoing retrieval (Fig. 1*D*).

## Materials and Methods

### Participants

A total of 46 participants were recruited from the National Institutes of Health (NIH) community and surrounding area. Three were excluded for excessive motion (≥5/8 task scans were excluded; see below, fMRI data analysis), one was excluded because of technical problems encountered while scanning, and two participants were excluded because of loss of verbal response data. The remaining 40 participants (23 female) had a mean age of 24.2 years (range: 20–34), were right-handed, had normal or corrected-to-normal vision, were native speakers of English, and reported no history of psychiatric or neurologic illness. Informed consent was obtained from all participants and the experiment was approved by the NIH Institutional Review Board (clinical trials number NCT00001360). Participants received monetary compensation for their participation. Other data from this sample, relating to effects of temporal distance on autobiographical recall in the hippocampus and neocortex, are described separately (Gilmore et al., 2020).

### Stimuli

Stimuli for the autobiographical recall and picture description tasks consisted of 48 photographic images depicting people participating in various activities. A subset of these stimuli were used in experiments reported by Gaesser et al. (2011) and Madore et al. (2014), while the remainder were newly acquired for this sample via internet search. Images were sized at 525 × 395 pixels (screen resolution: 1920 × 1080 pixels) and presented against a black background. Stimuli were presented using PsychoPy2 software (Peirce, 2007; RRID: SCR_006571) on an HP desktop computer running Windows 10.

Stimuli for the multicategory localizer task consisted of 120 images of eight different categories (abstract shapes, animals, body parts, static dots, faces, non-manipulable objects, scenes, phase-scrambled images, tools, and words) as well as a set of five images that instructed different movements. Stimuli were gray-scaled, sized at 600 × 600 pixels and taken from a larger collection described previously (Stevens et al., 2015). Localizer task stimuli were presented using the same software and hardware as the autobiographical recall and picture description stimuli.

### Autobiographical recall task

In this task, participants retrieved and described autobiographical memories in response to photographic cues. At the onset of each trial, an instruction screen directed participants to recall a specific event from one of three different recall periods (today, 6–18 months ago, 5–10 years ago), and provided participants with two different picture cues (Fig. 1). Participants had 11 s to select (via button press) the picture they preferred to use as an autobiographical memory cue. Two images were included to reduce the probability of event recall failure. Cues were rotated across conditions, although a subset was reserved for “today” recall periods that depicted more typical “everyday” scenes than were depicted in other images. The images were removed after a response was made, and at the end of the selection period an enlarged version of the selected image was presented in the center of the screen for 5 s. Participants were instructed during this time to use the picture to help think back to a specific event from the cued time period. Participants were further instructed to retrieve and describe a unique event for each trial (i.e., that they should not repeat event descriptions).

Immediately following the 5 s picture presentation, the image was removed and replaced with a white crosshair for 116.6 s. During this time, participants were instructed to describe the autobiographical memory with as much detail as possible for the full duration of the trial's narration period. In cases where participants stopped their narration early in the trial (e.g., with ≥20 s remaining), they were prompted by the experimenter. This took the form of the question “Are there any other details that come to mind?” as suggested by Levine et al. (2002), and participants heard the question via noise-cancelling headphones. Such prompts were rare (mean = 0.1 prompts/participant, SEM: 0.06). A stop cue, signaled by the white crosshair turning red for a period of 2.2 s, signaled the end of each trial. Participants therefore had an effective maximum description time of 118.8 s for each trial, although this frequently involved participants ending their narration before they were naturally done with an event's description. Trials were separated by 19.8 s of fixation, and three trials were included per scan run (one for each of the three temporal periods). Six autobiographical task runs were collected for each participant, and the order of time periods was counterbalanced across runs and participants. Participants were given practice with the task before scanning, and if the events described were not specific, participants were re-instructed and given further practice until specific episodes were being described. During this time, participants were also instructed that each described event should be unique (i.e., should only be described once in the experiment).

### Picture description task

This task required complex verbal descriptions in the absence of autobiographical recall. Instead, participants described the event being depicted in a cue photograph. The trial structure was the same as in the autobiographical recall task, and cues (excepting the “today” cues) were rotated pseudorandomly between tasks. Trials began with a cue/instruction screen that was accompanied by two images and participants had 11 s to select their preferred picture for that specific trial. Images were then removed until the end of the 11 s selection period, when an enlarged version of the selected image was centrally presented for 5 s. Participants were instructed to closely attend to the image so that in the following narration period, they could describe it such that someone who had not seen the image could understand what was being depicted. After the presentation period, a white crosshair was displayed for 116 s. During this time, participants described the image with as much detail as possible for the full duration of the narration period. As with the autobiographical recall task, a red fixation cross was displayed for 2.2 s at the end of the trial. Also consistent with the autobiographical recall task, participants were given a verbal cue if they ceased speaking early in a trial (mean = 0.35 prompts/participant, SEM: 0.12). Separation between trials and the number of runs per trial was identical to the autobiographical recall condition. Two runs of the picture description task were collected for each participant and their placement was counterbalanced across participants. As with the autobiographical task, participants were given practice before scanning to familiarize themselves with the nature of the picture description task and to address any questions the participant might have.

Participants were brought into the scanner after the initial task instruction period. Immediately following the experimental task scans, a high-resolution T1 was collected. Following the T1 scan, a resting-state scan of ∼8 min in length was collected in 32/40 participants, although these resting-state data are not used in this report.

### Multicategory localizer task

Approximately half of the participants (*N* = 22) returned for another scanning session to complete a prospectively chosen multicategory functional localizer task. This session always began with collection of a resting-state scan ∼8 min in length. For the localizer task, participants were presented with blocks of images from each included category and were directed to press a button when they noticed a repetition of the same image (or perform simple hand, toe, and tongue movements in the motor localizer block). Blocks were 22 s in duration and consisted of 20 images, each presented for 300 ms and separated by 800 ms of fixation. Blocks were separated by 11 s periods of fixation. The type of stimulus presented was counterbalanced across blocks and participants. Blocks contained one or two image repetitions, which were placed pseudorandomly.

### Audio recording and in-scanner speech

Participants spoke into a noise-cancelling, MR-compatible Optoacoustics FOMRI-III NC microphone (Optoacoustics Ltd.), connected to an M-Audio FastTrack Ultra 8-R USB audio/MIDI interface (inMusic). A Dell Precision M4400 laptop (Dell Inc.) recorded responses using Adobe Audition CS 6 (Adobe Inc.). Spoken audio recordings were transcribed for subsequent text analyses (see below, Transcript scoring). A parallel audio track captured a square wave pulse synchronized to the onset of each stimulus presentation to allow precise synchronization of audio tracks with the stimulus display, and thus with the BOLD timeseries.

Before beginning experimental scans, participants practiced speaking while the scanner was running. Real-time motion estimates were generated using a real-time AFNI implementation and the experimenter (A.W.G.) provided feedback to participants regarding the severity and types of motion that were being observed. The motion estimates included six parameters (three translational, three rotational) for each TR.

### Alignment of text, audio, and BOLD timeseries data

Before being transcribed, spoken audio tracks were processed in Audacity 2.3 (https://www.audacityteam.org/) to reduce residual background noise. Following transcription, the text for each track was checked against the original recorded audio to ensure that they were free of typographical errors. A Python-based text-to-speech alignment tool from the University of Pennsylvania Department of Linguistics (p2fa; Yuan and Liberman, 2008), was then used to provide timestamps for each word in each event transcript by aligning it with its corresponding audio track; outputs in alignment were manually edited to correct misalignments. Speech onset response times (RTs) for each event were calculated by comparing the time difference between the onset of the picture cue (recorded in a secondary audio track) and the onset of speech using MATLAB. The RT for each trial marked the onset of each spoken description, and as p2fa aligned all words with respect to the initial word, onset times and durations could be calculated for every spoken word in each description.

### Transcript scoring

Transcript contents were scored using an adapted version of the AI scoring system (Levine et al., 2002), modified to accommodate both memory and picture descriptions in a manner similar to that reported by Gaesser et al. (2011), with several additional modifications specific to this experiment. Briefly, the AI scoring procedure segments events into different details and classifies them as either “internal” (episodic details specific to the event being described) or “external” (details from unrelated episodes, semantic/non-specific statements, editorial comments, or repetitions of previously-described details). For picture description trials, details describing elements in the picture were considered to be internal, while inferences extending beyond what was shown were considered external. Consistent with guidelines outlined by Levine et al. (2002), the coder identified the “central” event for purposes of scoring if multiple events were described during autobiographical recall trials. One important update to the scoring procedure relates to the internal “event” details category. As described by Levine et al. (2002), these refer to a broad range of details including persons present, actions/reactions, weather conditions, and “happenings.” However, as it is known that different cortical regions support the processing of different concepts and object properties (Grill-Spector, 2003; Martin, 2016), the “event details” category was broken down into more specific detail types (e.g., person, object, activity). A full list of detail types is presented in Table 1. Each identified detail was associated with a single category that best represented its content.

**Table 1.** List of internal and external detail categories

Category*	Description
*Internal*	*Details associated with a spatially and temporally specific event*
Activity	Something done or undertaken by an individual or group of entities
Object	A non-living entity
Perceptual detail	Sensory details, including relative spatial positions and durations
Person	A human entity
Place	Description of the “where” of an event
Thought/emotion	Experienced and attributed emotional states, descriptions of ongoing thoughts
Time	A description of “when” an event took place, including time of day, season, etc.
Miscellaneous	Contains details that did not occur frequently enough for separate modeling, includes descriptions of animals, body parts, time, weather phenomena, etc.
*External*	*Details not specific to the reported event*
Episodic	Spatially and temporally specific details from occurrences other than the main event being described
Repetition	Repetitions of internal details
Semantic	General knowledge or background, often either tangential to or offered in support of the described event
Other	Editorial comments, banter, etc.

*Internal details are based on those initially described by Levine et al. (2002) and contain additional labels for specific conceptual categories, whereas external details are unchanged from those initially described.

Transcripts were scored by three separate raters, each of whom scored a subset of the overall participants. Raters were trained and their reliability assessed using independent pilot data. Reliabilities were calculated using intraclass correlation (ICC) analyses that employed a two-way random model. Overall, a strong reliability across all internal and external detail categories was observed, ICC(2,3) = 0.92. Focusing specifically on different internal detail categories revealed substantially similar results [all ICC(2,3)]: activity = 0.803, object = 0.439, perceptual = 0.979, person = 0.984, place = 0.925, thought/emotion = 0.933, time = 0.884, miscellaneous = 0.566. These values are comparable to those described previously (Levine et al., 2002).

Details were converted into event-related regressors for fMRI timeseries analysis (discussed further below, fMRI data analysis). The onset time began at the start of the first word for a given detail and lasted until the onset time of the next detail. Pauses between words were not modeled unless they were at least 4.4 s in duration, at which point they were treated as rest in the same manner as inter-trial fixation periods would be. In cases where several individual details of the same category were present, a single regressor was modeled for the combined duration of that detail category.

The average time per trial spent describing internal and external details was compared across autobiographical and picture description conditions using paired-sample *t* tests (two-tailed). Effect sizes were computed using G*Power (Faul et al., 2007; RRID: SCR_013726).

### fMRI data acquisition

Images were acquired on a General Electric Discovery MR750 3.0T scanner, using a 32-channel phased-array head coil. Functional images were acquired using a BOLD-contrast sensitive multi-echo echo-planar sequence [Array Spatial Sensitivity Encoding Technique (ASSET) acceleration factor = 2, TEs = 12.5, 27.7, and 42.9 ms, TR = 2200 ms, flip angle = 75°, 64 × 64 matrix, in-plane resolution = 3.2 × 3.2 mm]. Whole-brain EPI volumes (MR frames) of 33 interleaved, 3.5-mm-thick oblique slices were obtained every 2.2 s. Slices were manually aligned to the AC-PC axis. A high-resolution T1 structural image was also obtained for each subject (TE = 3.47 ms, TR = 2.53 s, TI = 900 ms, flip angle = 7°, 172 slices of 1 × 1 × 1 mm voxels) after the collection of task data.

Foam pillows were provided for all participants to help stabilize head position and scanner noise was attenuated using foam ear plugs and a noise-cancelling headset. This headset was also used to communicate with the participant during their time in the scanner. Heart rate was recorded via a sensor placed on each participant's left middle finger and a belt monitored respiration for each participant.

### fMRI preprocessing

fMRI data were preprocessed using AFNI (Cox, 1996; RRID: SCR_005927) to reduce noise and facilitate across-subject comparisons. Initial steps included a removal of the first four frames of each run to remove potential T1 equilibration effects (3dTcat), despiking to remove large transients in the timeseries (3dDespike), and framewise rigid-body realignment to the first volume of each run (3dvolreg). Following these initial steps, data from the three echoes acquired for each run were used to remove additional noise using multi-echo independent components analysis (ME-ICA; Kundu et al., 2012, 2013, 2017). This procedure initially calculates a weighted average of the different echo times to reduce thermal noise within each voxel, and then uses spatial ICA and the known properties of T_2_* signal decay over time (and thus, over echoes) to separate putatively neural components from artefactual components, such as thermal noise or head motion (Power et al., 2018). To be retained, components must show a strong fit with a model that assumes a temporal dependence on signal intensity and also a poor fit with a model that assumes temporal independence (Kundu et al., 2012). Selection criteria were left at the default settings of AFNI's *tedana.py* function. Following ME-ICA processing, data from each scan run were aligned across runs, registered to each individual's T1 image, and then resampled into 3-mm isotropic voxels and linearly transformed into Talairach atlas space (Talairach and Tournoux, 1988).

### fMRI data analysis

#### Autobiographical recall and picture description modeling

All task scans consisted of 210 MR frames (214 before initial frame discarding) and lasted 7 min, 51 s in duration. Six autobiographical retrieval and two picture description runs were collected for each participant. Average run-level motion estimates were derived using AFNI's @1dDiffMag based on three translational and three rotational motion parameters; runs with >0.2 mm/TR were excluded. As noted under Participants, this resulted in the exclusion of three participants because of excessive motion. Additionally, two autobiographical task runs were excluded from four additional participants and one autobiographical task run was excluded from five participants. Over 94% of scans involving speech were therefore retained, and mean motion estimates for each condition fell considerably below the 0.2 mm/TR cutoff (autobiographical recall: 0.125 ± 0.031 mm/TR; picture description: 0.120 ± 0.030 mm/TR). Thus, speech-related motion did not seem to cause a widespread exclusion of data despite almost 6 min of time spent speaking in each task scan.

Before analysis, functional data from each subject were smoothed using a 3-mm full-width at half-maximum (FWHM) Gaussian kernel to account for inter-subject anatomic variability. Each voxel was normalized by its mean signal on a runwise basis and detrended to account for scanner drift effects with first-order polynomials. Analyses were based on a general linear model (GLM) approach (3dDeconvolve). Data from each time point were treated as the sum of all effects present at that time point. Analyses were conducted as a mixed block/event related design, and two sets of GLMs were created. Effects were modeled by convolving an HRF with a boxcar (via AFNI's “BLOCK” response model) set to an appropriate length, as described below.

#### Combined autobiographical and picture description GLMs

The first set of GLMs used data from both autobiographical recall and picture description trials and was used in the comparison of their sustained effects (described below). The picture selection phase for all trial types was modeled using a single regressor with a duration of 11 s. The picture display phase for all trials was also modeled using a single regressor with a duration of 5 s. Four regressors, each with a duration of 118.2 s, modeled activity associated with the speaking period of each autobiographical recall condition (today, 6–18 months ago, 5–10 years ago) and the picture description condition. Effects associated with each category of internal and external detail (listed in Table 1) were modeled across both the autobiographical recall and picture description conditions (i.e., there was a single “place” regressor that accounted for all instances in which a place detail was described in either of the task conditions) with the spoken duration of each detail included as a duration modulator via AFNI's dmBLOCK function. Finally, six motion parameters (three translational, three rotational) were included in each subject's GLM as regressors of non-interest.

#### Autobiographical recall GLMs

Following the initial comparison of the autobiographical and picture description tasks, a separate set of GLMs was constructed to specifically examine reactivation effects during recall. This second set of GLMs was constructed identically to the first, except that it only included autobiographical recall runs.

#### Multicategory localizer

Category-selective cortex was identified using independent localizer data collected from 22 of the sample's 40 participants. Each localizer run was 174 MR frames in length (initially 178) and lasted for ∼6 min, 23 s. Six localizer task scans were collected from each participant and no scans were dropped for motion or other causes. Preprocessing of localizer data followed the same steps previously described. Analyses were again based on a GLM approach in which activity for each block type was modeled using an HRF convolved with a 22 s boxcar, consistent with the length of each trial block. Separate regressors coded for each of the 11 stimulus categories included. Motion related regressors were also included in each subject's GLM as described previously.

### Differences in sustained activity associated with autobiographical recall and picture description trials

Sustained activity associated with the autobiographical recall and picture description conditions was compared using paired-samples, two-tailed *t* tests. Activity associated with autobiographical recall trials was averaged across all three recall periods and contrasted against activity associated with the picture description task. In a separate analysis, only activity from the autobiographical recall “today” time period was compared with the picture description tasks. Statistical images were corrected for multiple comparisons to achieve a whole-brain *p* < 0.05. This correction approach was conducted using Monte Carlo simulations performed in 3dClustSim (Cox et al., 2017a,b) which specified a voxelwise significance threshold of *p* < 0.001 and a minimum cluster extent (*k*) of 18 voxels. A similarity comparison was then made by binarizing each mask and assessing their overlap. A Dice similarity coefficient was computed to assess the agreement of the two maps, with the requirement that overlapping voxels needed to have the same sign in both maps (i.e., both had to have a significant positive *t* value or both had to have a significant negative *t* value to be considered intersecting in the Dice calculation).

For purposes of visualization, statistical maps were sampled from the volume to a partially-inflated representation of the cortical surface using Connectome Workbench software (Marcus et al., 2011). All coordinates listed in this report have been converted to MNI152 space.

### Comparing BOLD activity during verbal detail descriptions

Autobiographical reactivation effects associated with descriptions of internal details were of primary interest in this report. An important step in this process was determining which categories should be examined. The selection process first involved sorting each detail category by the total amount of time spent describing it across autobiographical recall trials, and we then selected the most common categories that could be grounded in the independent multicategory localizer task. Activity details (which involved the movements or actions of entities) were the most common, followed by perceptual details, and then place, object, and person details. The perceptual details category was excluded from analysis because of the variable nature of its contents, which encompassed sensory details across multiple modalities as well as more general information regarding durations and relative spatial locations (Table 1; see also Levine et al., 2002). Activity associated with the activity, place, object, and person detail descriptions was thus examined at a voxelwise level across the whole brain. Two of these categories were social in nature (activity, person) and two were non-social (place, object). To identify reactivation effects associated with each detail type, each “social” detail category was contrasted with an average of the non-social detail types (e.g., person activity was compared with the mean activity associated with place and object details), and each non-social detail was compared with the average activity of the two social details (e.g., place-related activity was compared with the mean of activity and person details). Contrasts were conducted using paired samples, two-tailed *t* tests and each was corrected for multiple comparisons to achieve a whole-brain *p* < 0.05 (voxelwise *p* < 0.001; *k* ≥ 18).

### Overlap of category-selective cortex derived during verbal description periods and the multicategory localizer

Contrasts of localizer data were constructed to provide analogues for each of the detail reactivation effects identified in the autobiographical recall data, so that these could be grounded to within-sample functional neuroanatomy. Recalled places were matched with visually presented scenes and recalled objects were matched by averaging the three types of objects present in the localizer (abstract objects, non-manipulable objects, tools). As the person and activity reactivation maps were quite similar and both reflected social aspects of events, BOLD activity associated with these detail types was combined and compared with a map of task-negative localizer task responses. This choice reflects the fact that many regions across the default network, itself first identified based on task-induced deactivations (Shulman et al., 1997), are consistently associated with social cognition and have been referred to as the “social brain” system (Martin and Weisberg, 2003; Buckner et al., 2008; Frith and Frith, 2010; Mars et al., 2012; Jack et al., 2013; Stanley and Adolphs, 2013; Wang and Olson, 2018).

Each localizer contrast was conducted using paired samples, two-tailed *t* tests. Scene-selective activity was defined by a contrast of scene blocks with an average of object and face blocks; object-related activity was defined by contrasting objects with faces and scenes, and task-negative responses were defined by a one-sample test across all block types versus baseline. Each statistical map was corrected to a whole brain *p* < 0.05 level by requiring a voxelwise significance of *p* < 0.001 and cluster *k* ≥ 21 voxels.

The extent of overlap between clusters preferentially associated with the recall of each detail type (place, object, person, and activity) and their localizer analogues was quantified using a conjunction image approach. Significant positive voxels/clusters in each map were converted to binary masks and subsequently summed. As masks were typically of uneven voxel counts, the proportion of the smaller contained within the larger map was quantified to provide a descriptive index of the degree to which reactivation effects manifested in independently localized, category-selective cortex. A separate quantitative description was provided by calculating the Dice similarity coefficient (DSC) for each map and comparing it to a null model, as will be described below.

### Creation and use of synthetic data as a comparison for reactivation effects

To empirically determine the significance of each Dice coefficient, simulations were performed using simulated data. First, 3dClustSim was used to randomly generate 6000 maps that were matched in smoothness to the autobiographical recall data (2000 were then assigned to each detail type of interest: place, object, social). Another 6000 were generated that were matched in smoothness to the Localizer data. The masks associated with each condition were then thresholded such that only the top *N* voxels were retained, where *N* = the number of significant voxels in each source mask. For example, there were 1608 voxels in the autobiographical recall “place” detail map, and so for its randomly generated associate maps, the 1608 voxels with the highest value were retained and subsequently binarized. This process was repeated for the recall object and recall social maps, as well as the three localizer maps (scene, object, and task-negative).

Dice coefficients were then calculated for the synthetic versions of each recalled detail type and each localizer analogue (place/scene, object/object, social/task-negative). At random, a map from each pool was selected and their Dice similarity was compared, and the process was repeated 2000 to build a null distribution. The significance of each observed Dice coefficient in the real fMRI data were then computed by identifying its rank-ordered location in the null distribution and dividing by 2000.

To establish that overlap was not just greater than would be expected by chance, but also specific to each recalled detail type, the Dice coefficients were calculated for all recall-localizer comparisons. The resulting 3 × 3 matrix therefore reflected the degree of overlap for within-category and across-category comparisons.

## Results

### Typical autobiographical recall activations are observed when ignoring event details

Before examining dynamic retrieval effects, it was important to demonstrate that regions typically associated with autobiographical recall could be observed when the present data are analyzed in a manner consistent with prior literature, that is, without taking verbal descriptions into account. Sustained activity was therefore compared between the autobiographical recall speaking periods and picture description task speaking periods. Participants spent almost the entirety of each narration period speaking, regardless of condition, averaging 111.1 s per trial in the case of autobiographical recall trials and 105.7 s for the picture description trials. This difference, while numerically and proportionally small, was significant, *t*_(39)_ = 3.78, *p* < 0.001, *d* = 0.598. Whereas the autobiographical narration periods should be associated with activity in regions typically associated with autobiographical memory (and based on differences in overall speaking time or in effort associated with narrating contents in memory, perhaps also regions associated with speech production), regions associated with the picture description task should likely fall in locations associated with visual working memory or attentional control (Courtney et al., 1998; D'Esposito and Postle, 2015), as the latter task required rapidly encoding, maintaining, and attending to details of a complex visual scene throughout each narration period.

**Figure 2.**
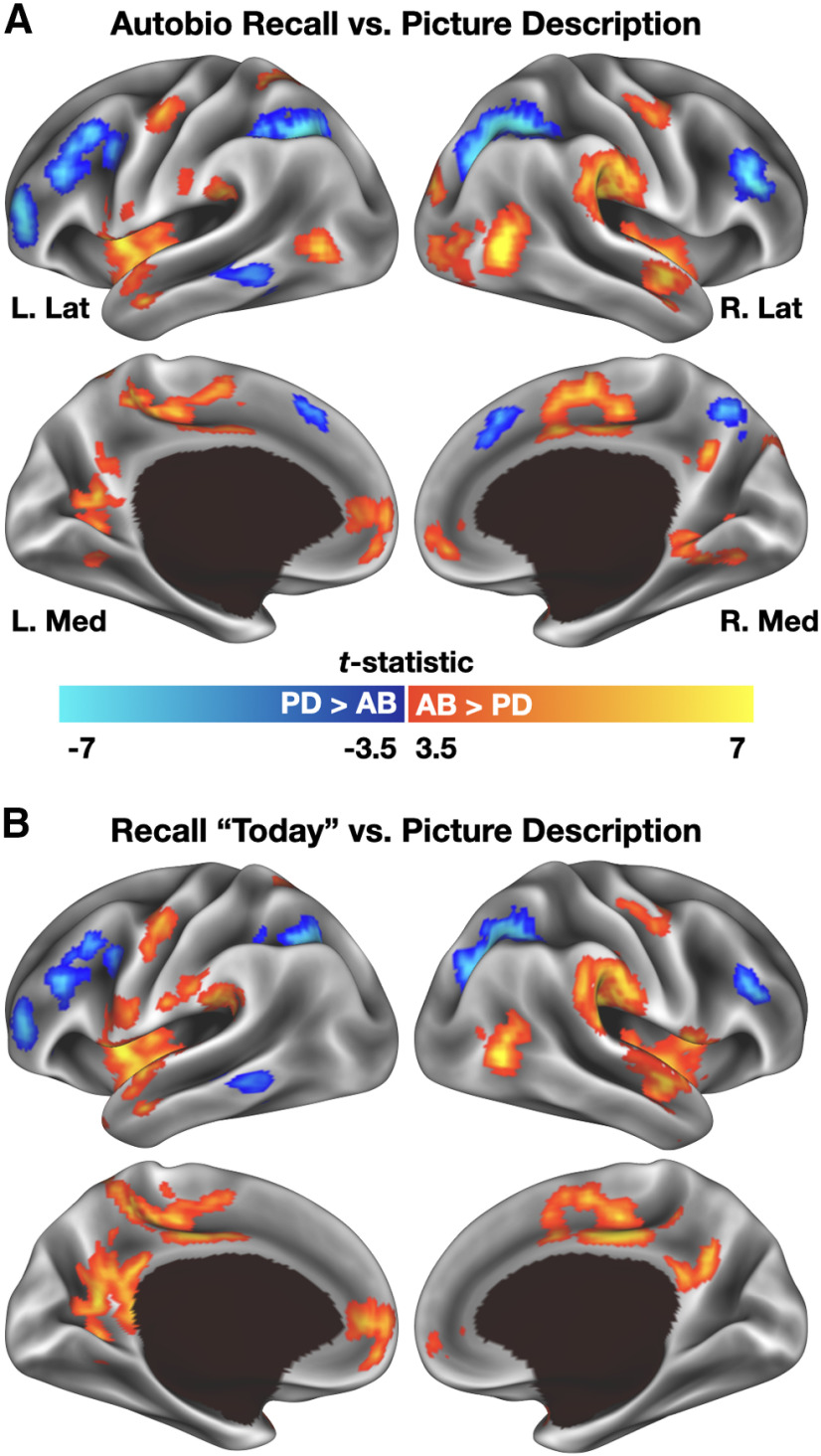
Contrast of sustained BOLD activity associated with autobiographical recall and picture description trials. ***A***, Autobiographical recall trials elicited greater activity than picture description trials bilaterally in anterior superior temporal gyrus and superior temporal sulcus, ventral medial parietal cortex, and medial prefrontal cortex, among other regions. Picture description preferentially engaged the intraparietal sulcus bilaterally, as well as dorsal and ventral lateral prefrontal regions. ***B***, Repeating the analysis using only a subset of the autobiographical recall trials, those associated with recall of events from earlier on the day of scanning, produced the same overall pattern of results. For display purposes, voxelwise statistical maps were projected onto a cortical surface using Connectome Workbench software (Marcus et al., 2011). PD, picture description; AB, autobiographical recall.

Comparing sustained activity between tasks (paired samples *t* test, two-tailed) identified numerous regions of difference across the cortex, despite each period extending almost 2 min in duration (Fig. 2*A*; Table 2). For autobiographical recall, these included medial prefrontal and posterior cingulate cortex, bilateral parahippocampal cortex, and bilateral anterior superior temporal gyrus (all of which are commonly associated with autobiographical recall; Svoboda et al., 2006; Boccia et al., 2019), as well as the left mid-insula and bilateral aspects of somato-motor cortex and the paracentral lobule (presumably reflecting the slightly greater overt speech production during autobiographical trials; Dronkers, 1996; Gracco et al., 2005). In contrast, regions preferentially engaged by the picture description task included dorsal and anterior lateral prefrontal cortex and the intraparietal sulcus. Clusters associated with the picture description task therefore appear to coincide with regions associated with visual working memory maintenance (Courtney et al., 1998; Ranganath and D'Esposito, 2005; D'Esposito and Postle, 2015) or attentional control (Dosenbach et al., 2006, 2008).

**Table 2.** Regions displaying significantly different sustained activity during autobiographical recall and picture description

Region	X	Y	Z	*k*
*Autobiographical recall* > *picture description*				
Left supplementary motor area/cingulate cortex	−1	−20	47	218
Right supramarginal gyrus/superior temporal gyrus/posterior insula	62	−31	28	183
Left insula	−43	2	−3	143
Right insula/anterior superior temporal sulcus	50	−6	−12	140
Right posterior middle temporal gyrus/lateral occipital cortex	43	−71	2	123
Ventromedial prefrontal cortex	2	56	−6	88
Left ventral parieto-occipital sulcus/posterior parahippocampal cortex	−20	−58	5	43
Left anterior superior temporal gyrus	−59	1	−16	31
Dorsal anterior cingulate cortex	2	29	44	31
Right posterior parahippocampal cortex	24	−56	−9	28
Right precentral gyrus	47	−9	47	27
Right cuneus	18	−85	24	26
Left posterior middle temporal gyrus/lateral occipital cortex	−43	−71	2	22
Left superior temporal gyrus/posterior insula	−56	−35	21	22
Posterior cingulate cortex	2	−60	26	22
Left precentral gyrus	−43	−9	50	20
*Picture description* > *autobiographical recall*				
Right intraparietal sulcus	34	−59	40	178
Left intraparietal sulcus	−33	−59	43	125
Left dorsolateral prefrontal cortex	−46	23	31	115
Left ventrolateral prefrontal cortex	−40	48	5	84
Right lateral prefrontal cortex	47	31	23	58
Left posterior inferior temporal gyrus	−59	−50	−9	46
Right dorsal precuneus	12	−65	44	23

Coordinates reflect centers of mass in MNI152 space.

Autobiographical recall trials were cued memories from three different time periods (today, 6–18 months ago, and 5–10 years ago). It was therefore possible that the results depicted in Figure 2*A* could result from differences in temporal distance between remote recall periods and the picture description task. To better match the temporal distance of retrieved episodes, minimize potential consolidation effects related to time or sleep (McClelland et al., 1995; Klinzing et al., 2019), and equate total trials per condition, a more restricted analysis was conducted. In this follow-up, only autobiographical recall trials from the “today” recall period were compared with picture description trials. Results were little changed when compared with the prior analysis (Fig. 2*B*). The Dice similarity coefficient of the thresholded maps was fairly high, DSC = 0.63, suggesting strong consistency regardless of whether one includes all autobiographical recall trials or only a “best equated” subset. Thus, when ignoring dynamic activity related to detail retrieval, overt autobiographical recall conditions can be shown to activate regions consistent with those reported in previous studies of autobiographical memory retrieval (Svoboda et al., 2006; Chen et al., 2017a).

### Autobiographical descriptions consisted mainly of internal details, often relating to physical entities and perceptual information

**Figure 3.**
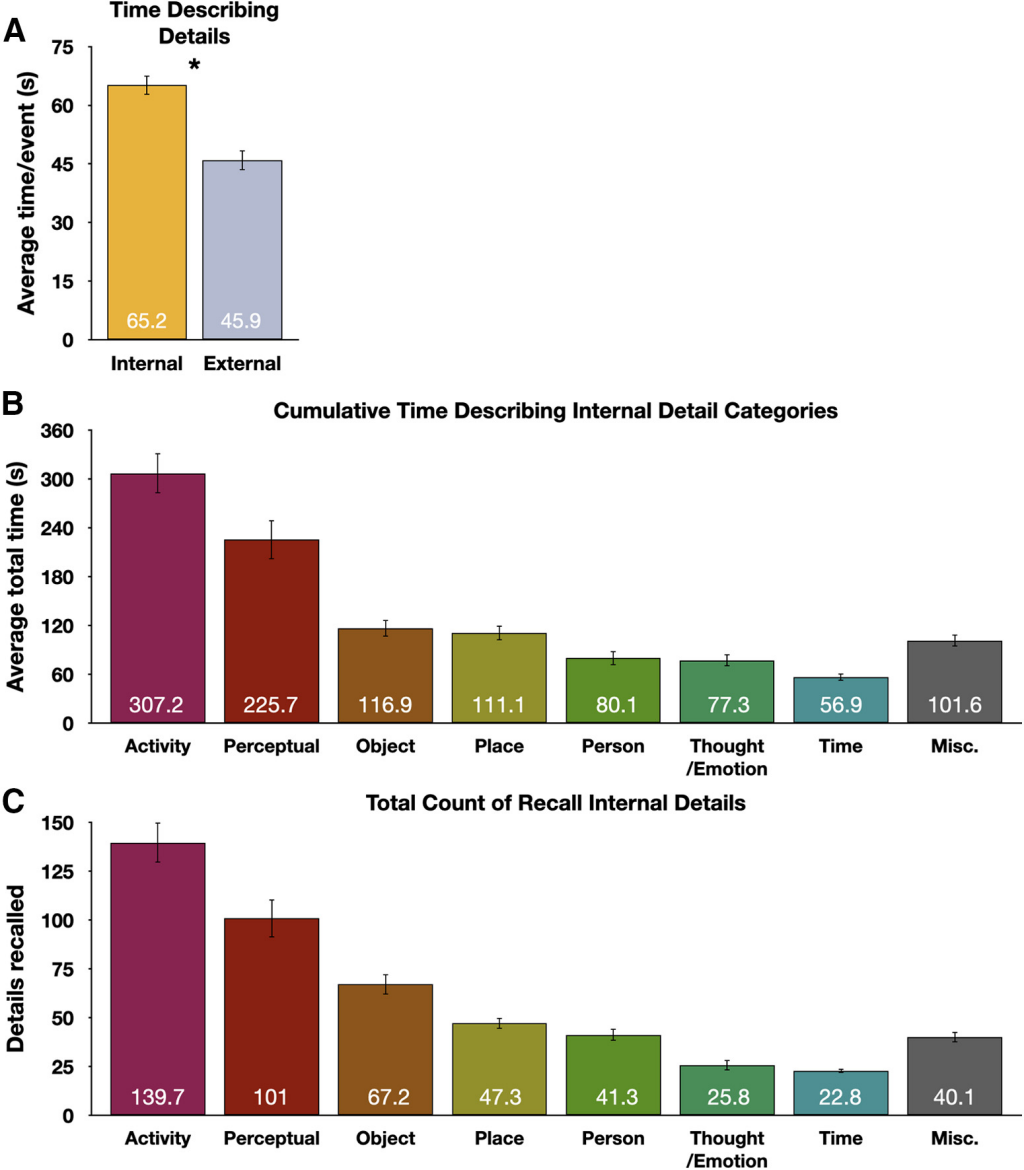
Time spent describing events and details. ***A***, Participants spent significantly more time per event describing internal than external details. ***B***, Sorting internal detail categories by cumulative time spent describing them revealed that participants spent most of the time describing activities that occurred, perceptual details, or the objects, places, and people associated with each event. ***C***, Tabulating a count of average times each detail type was recalled produces the same overall pattern of results. Error bars denote *SEM*.

Verbal reports were recorded, transcribed, and the text was synchronized with the audio so that each word had an onset time and duration. Reports were scored for content using an adapted version of the AI (Levine et al., 2002; Gaesser et al., 2011). Summarized briefly, event descriptions can be divided into discrete elements, or “details,” and each can be classified as internal (episodic and specific to the event being recalled), or external (not specific to the event being described, or a repetition of a previously-described detail). Internal and external details can then be further broken down into specific subtypes, such as those involving a person or a perceptual detail (listed in Table 1; for in-text examples, see Fig. 1*B*). Participants spent significantly more time describing internal details than external details, *t*_(39)_ = 4.00, *p* < 0.001, *d* = 0.632 (Fig. 3*A*). Across all recalled events, participants spent ∼18 min describing internal details.

To better characterize the types of internal details provided, time spent describing each internal category was tabulated across all autobiographical recall trials. Participants spent the most time describing activity details (descriptions related something performed or undertaken by an individual or group of entities), followed by perceptual details (those related to sensory details, descriptions of durations, and relative spatial positions), and then the detail categories of object, place, and person (Fig. 3*B*). In descending order after these types were details related to thoughts/emotions and to time. A catch-all “miscellaneous” bin consisting of internal details that did not fit into other categories was present but is not further discussed in this report. Thus, the main contents of descriptions were weighted toward physical entities, their ongoing activities, and their appearances. This basic pattern was maintained if one tabulates the total number of details recalled for each internal category instead of looking at the total duration (Fig. 3*C*).

### Differential BOLD activity was associated with verbal descriptions of activities, people, places, and objects

**Figure 4.**
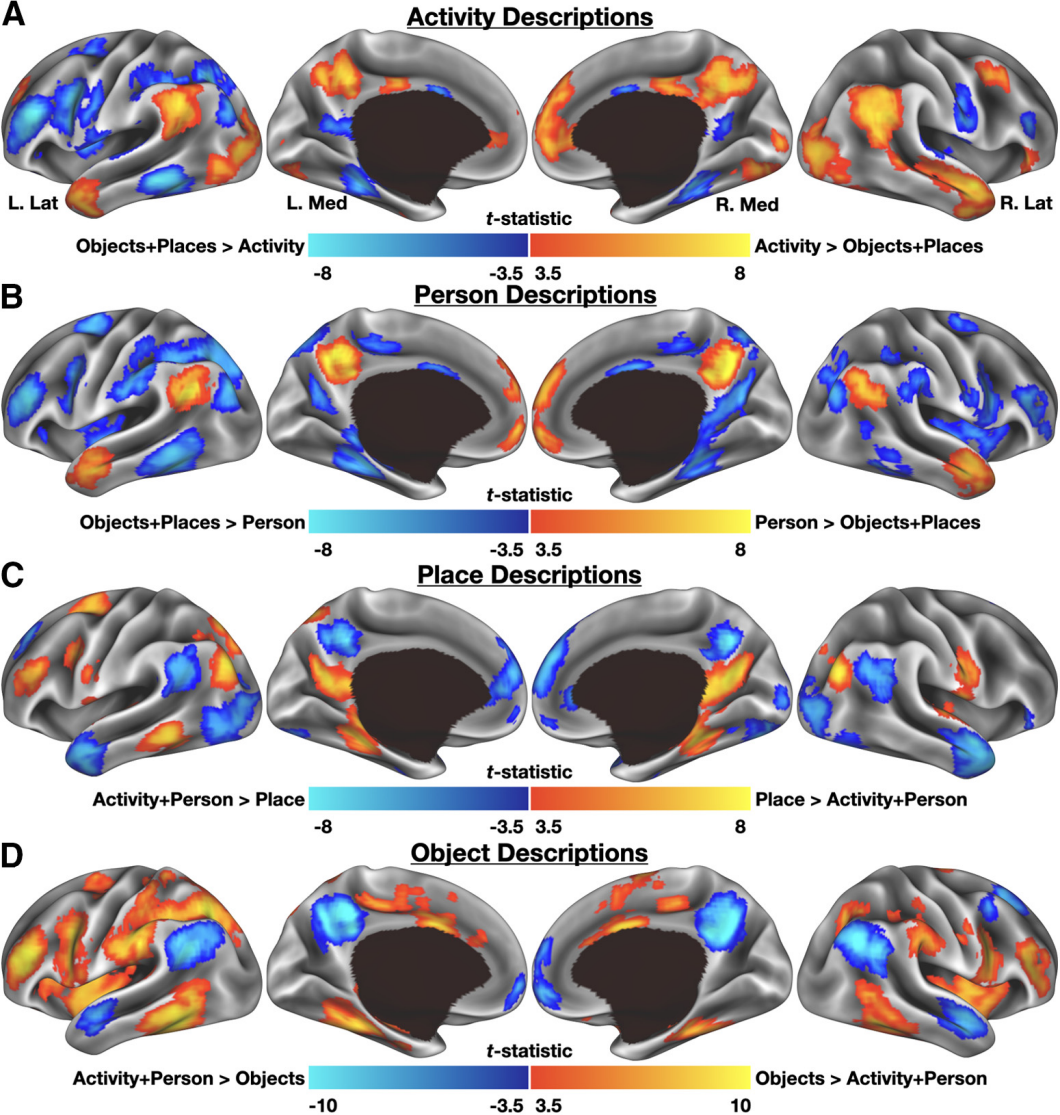
Differences in BOLD activity associated with the verbal description of autobiographical internal details. ***A***, Activity details preferentially engaged regions of medial prefrontal cortex, posterior cingulate cortex, the anterior temporal lobes, and posterior superior temporal sulcus/angular gyrus. ***B***, Describing person details preferentially engaged similar regions of cortex. ***C***, Place details bilaterally engaged parahippocampal cortex, the posterior angular gyrus and retrosplenial cortex/parieto-occipital sulcus. ***D***, Describing objects preferentially engaged a large expanse including the lateral occipital complex, dorsal parietal cortex, and somato-sensory cortex.

With knowledge of what was being described and when, it was possible to look for reactivation effects associated with the naturalistic retrieval of different types of details. Four of the five most common internal detail categories were targeted for further investigation: activity, place, object, and person (see Materials and Methods). Despite detail descriptions ranging from under a second to tens of seconds in duration (e.g., object detail durations ranged from 0.06 to 28.9 s with a median duration of 1.52 s), and despite being based on a scoring metric (the AI) that was designed for use behaviorally and not with fMRI data, results suggest that detail retrieval reliably produces reactivation effects specific to each recalled category. Reactivation effects associated with activity details were generally observed bilaterally and included regions of occipital cortex, anterior temporal lobe, posterior cingulate cortex, dorsomedial prefrontal cortex, and angular gyrus/posterior superior temporal sulcus (especially on the right hemisphere; Fig. 4*A*). Reactivation effects associated with person details appeared in many similar locations to those of activity details and were observed in regions of ventromedial prefrontal cortex, posterior cingulate cortex, the left angular gyrus/posterior superior temporal sulcus, and bilaterally in anterior aspects of the superior and middle temporal gyri and superior temporal sulcus (Fig. 4*B*). Reactivation effects associated with places were observed bilaterally in parahippocampal cortex, the posterior angular gyrus, and retrosplenial cortex/parieto-occipital sulcus, as well as left superior and middle frontal gyri (Fig. 4*C*). Effects associated with object details were more broadly observed, including in lateral and ventral occipital cortex, dorsal and anterior parietal cortex, somatosensory cortex (particularly on the left hemisphere), and several regions in anterior frontal cortex and the inferior frontal junction (Fig. 4*D*).

### Reactivation effects overlapped with independently defined category-selective cortex

Regions associated with detail reactivation effects resemble the distributed networks thought to represent social phenomena, places, and objects (Martin and Chao, 2001; Grill-Spector, 2003), but this similarity would be more convincing if it were demonstrated empirically within the same group of participants. A multicategory functional localizer task was prospectively selected to provide independent means of identifying category-selective cortex in this experiment. The localizer was collected for approximately half of the participants (*N* = 22) in a separate scanning session (for details, see Materials and Methods). Place and object detail types had clear analogues in the localizer data and were paired accordingly: internal place details were matched with visually presented scenes and internal object details were matched with visually presented objects. A map of task-induced deactivations across all localizer blocks was used as a comparison for activity and person details; this contrast was intended to capture canonical default network regions (Shulman et al., 1997) as these have been strongly associated with social processing (Martin and Weisberg, 2003; Mars et al., 2012; Jack et al., 2013; see also Buckner et al., 2008; Frith and Frith, 2010; Stanley and Adolphs, 2013; Wang and Olson, 2018) and both the activity and person categories involve social aspects of an experience. Activity and person details were combined before assessing overlap with the task-negative map. The localizer-defined maps for each category were corrected for multiple comparisons, binarized, and the overlap with their respective internal detail analogues was assessed (Table 3). Similarity between maps was measured using a Dice similarity coefficient. Each coefficient was then compared with 2000 simulated overlaps using synthetic data matched for smoothness and areal extent to each real map (see Materials and Methods).

**Table 3.** Regions of overlap between overtly described autobiographical detail categories and localizer-defined category-selective cortex

Region	X	Y	Z	*k*
*Place-scene overlap*				
Right parahippocampal cortex/anterior parahippocampal place area	28	−37	−13	95
Left parahippocampal cortex/anterior parahippocampal place area	−27	−37	−13	87
Left posterior angular gyrus/anterior occipital place area	−36	−82	27	79
Right parieto-occipital sulcus/retrosplenial complex	18	−54	12	70
Right posterior angular gyrus/anterior occipital place area	37	−79	27	33
Left parieto-occipital sulcus/retrosplenial complex	−14	−54	12	25
*Object-object overlap*				
Left inferior temporal gyrus/lateral occipital cortex	−49	−59	−9	148
Left medial fusiform gyrus	−30	−41	−23	23
Left anterior supramarginal gyrus	−59	−27	37	20
Left anterior intraparietal sulcus	−46	−33	45	6
Left postcentral gyrus	−43	−38	62	5
Left postcentral gyrus	−24	−54	63	2
*Social processing-task-negative overlap*				
Posterior cingulate cortex	2	−56	33	401
Medial prefrontal cortex	5	57	11	311
Right anterior middle/superior temporal gyrus	56	0	−26	279
Left anterior middle/superior temporal gyrus	−56	0	−26	203
Left angular gyrus/posterior superior temporal sulcus	−49	−56	29	188
Right angular gyrus/posterior superior temporal sulcus	50	−62	33	131
Right superior frontal gyrus	21	39	47	58
Left cerebellum (lobule VIIa)	−30	−80	−34	29
Right cerebellum (lobule VIIa)	28	−80	−34	14
Right middle frontal gyrus	40	14	41	11
Left middle temporal gyrus	−53	−37	−3	7
Right superior frontal gyrus	15	33	54	4
Right anterior superior temporal gyrus	53	19	−27	3

Coordinates reflect centers of mass in MNI152 space.

**Figure 5.**
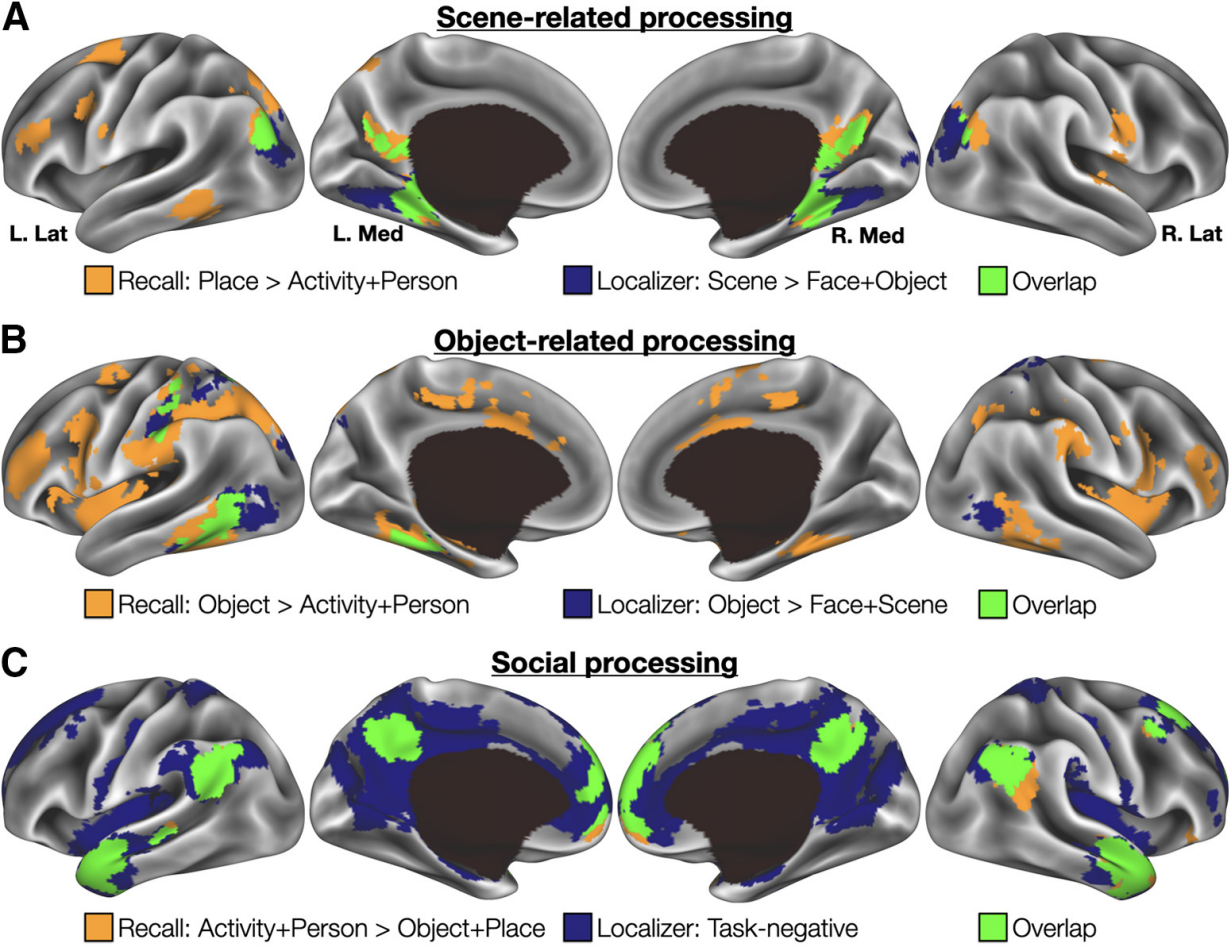
Overlap of localizer-defined category-selective cortex and reactivation effects associated with autobiographical detail descriptions. ***A***, Overlap of place details and scene-selective cortex was observed in bilateral parahippocampal cortex, posterior angular gyrus, and retrosplenial cortex/parieto-occipital sulcus. ***B***, Object-related overlap was located most prominently in lateral occipital cortex. ***C***, Descriptions of social details fell almost entirely within a “task-negative” map derived from the Localizer task.

**Figure 6.**
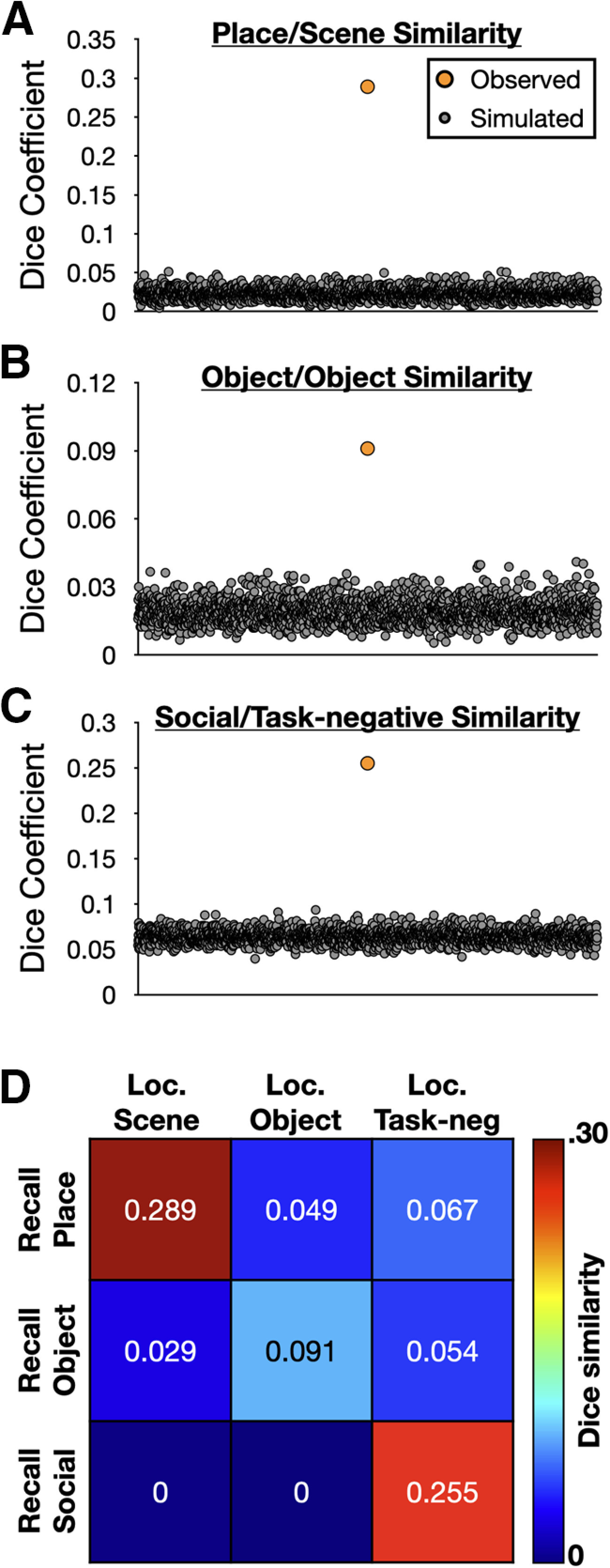
Recall-localizer map consistency was significantly greater than would be expected by chance and was category-specific. The Dice coefficient between (***A***) recalled place and localizer scene maps, (***B***) recalled object and localizer object maps, and (***C***) recalled social detail and localizer task-negative maps, was never equaled or exceeded by synthetic maps matched for each condition's smoothness and voxel extent. A total of 2000 simulated maps were generated for each condition and used to empirically determine the significance of each obtained Dice similarity. ***D***, An adjacency matrix of all recalled detail categories (rows) with each localizer condition (columns) indicated that overlap was always greatest between the nominally matched recall and localizer conditions, with off-diagonal Dice coefficients being reduced or zero in magnitude.

For place/scenes, overlap occurred in retrosplenial cortex and the parieto-occipital sulcus, parahippocampal cortex, and posterior angular gyrus (or, respectively, retrosplenial complex, anterior parahippocampal place area, and anterior occipital place area, to use different terminology for the same regions; Fig. 5*A*). Furthermore, the overlap was configured such that recall effects tended to extend anteriorly from the overlap, and Localizer effects posteriorly, consistent with recent “anterior-posterior” differentiations of mnemonic and perceptual categories in scene-selective cortex (Baldassano et al., 2016; Silson et al., 2019a). Quantitatively, ∼36% of the localizer-defined scene-selective cortex fell within the internal place map (389/1085 voxels). This overlap produced an overall *DSC* = 0.289 (*p* = 0.0005); the similarity was never exceeded in comparison simulations (Fig. 6*A*).

Object/object overlap occurred most prominently in the left inferior temporal gyrus/lateral occipital cortex, as would be expected for category-specific reactivation effects involving objects (Malach et al., 1995; Martin et al., 1996; Fig. 5*B*). Additional overlap was present in the medial fusiform and left anterior supramarginal gyrus/somatosensory cortex. 32% of the localizer-defined object-selective cortical map fell within the internal object map (205/638 voxels), producing a modest but significant overall *DSC* = 0.091, *p* = 0.0005. As with the place-scene value, this was greater than any similarity observed in simulation data (Fig. 6*B*).

Locations associated with “social” (activity and person) details fell overwhelmingly in the task-negative localizer map (75%, 1641/2196 voxels; Fig. 5*C*). Notable locations of overlap included ventromedial prefrontal cortex, posterior cingulate cortex, and bilaterally the angular gyrus, anterior middle temporal gyrus, and inferior temporal gyrus. The overall similarity was again relatively high (*DSC* = 0.255, *p* = 0.0005) despite the size disparity between the two source maps (2196 vs 10 846 voxels), and no simulated data reached or exceeded this value (Fig. 6*C*).

Having established that each overlap was significantly greater than could be expected by chance, a final question was to compare similarity across, rather than within, conditions. This was necessary to demonstrate that reactivation effects were not only significant, but also specific to each condition. A similarity matrix was therefore constructed to compare all recall and localizer maps (Fig. 6*D*), and the on-diagonal values always exceeded off-diagonal values. Thus, dynamic effects were both highly associated with category-selective cortex and are more similar to their own category than other categories examined in this report.

## Discussion

We used naturalistic in-scanner verbal recall to investigate vivid event retrieval, focusing in particular on how the brain reactivates details throughout a prolonged recall period. Results demonstrated that, if one ignores the moment-to-moment content and focuses only on sustained activity, a “standard” map of regions associated with autobiographical recall is observed. However, by specifically focusing on different types of details as they are naturally recalled and described, one instead can identify transient, detail-specific reactivation effects that are grounded in regions of cortex thought to represent relevant category-level information. These data speak to basic questions about how humans can engage in vivid mental time travel to re-experience past events through reconstructive uses of memory.

### Sustained activity links current and prior autobiographical retrieval results

When focusing on sustained, rather than transient, effects associated with the autobiographical recall and picture description tasks, it was possible to capture regions typically associated with mental time travel (Fig. 2). This analysis demonstrated that overt autobiographical recall can replicate prior results obtained using covert recall (for a recent meta-analysis, see Boccia et al., 2019) and therefore elaborate on a well-established literature. Indeed, the observation that sustained effects in this experiment identify regions previously associated with autobiographical memory further reinforces the central role that default/core regions play in supporting mental time travel (Schacter et al., 2007, 2012; Buckner et al., 2008), given the long duration of each trial.

Importantly, while overt recall can recapitulate effects observed in covert recall, the converse is impossible, one cannot observe dynamic effects if one does not look for them. Broader adoption of overt recall strategies will be critical to further understand how the brain supports episodic autobiographical recall or simulations of hypothetical events.

### Capturing dynamic reactivation effects

Inclusion of overt recall provided insights into the dynamic, ongoing retrieval process as participants recalled and described complex events. A formalized approach was necessary to identify and quantify verbalized content. One possibility would be to categorize every single word for each event, but a well-researched alternative already existed in the form of the AI. The AI was designed as an instrument to separate episodic and non-episodic details associated with the recall of specific episodic and autobiographical memories (Levine et al., 2002). It is often employed to study differences across age groups (Gaesser et al., 2011; Willoughby et al., 2012), psychiatric conditions (Söderlund et al., 2014), cognitive training strategies (Madore et al., 2014), and neurodegenerative disorders (Addis et al., 2009b; Irish et al., 2018). The AI has been used when studying brain-behavior relationships using covert retrieval (St. Jacques et al., 2012; Palombo et al., 2016), and in such cases was applied to out-of-scanner verbal reports.

The present use of AI thus extends considerably past its original use parameters, but results suggest that it could be adapted effectively. Indeed, each analyzed detail type produced reliable, content-specific activation patterns (Fig. 4). One crucial adaptation to the AI used here was to divide the broad internal event detail category into distinct subcategories. Event details were originally constructed to reflect a diverse range of spoken details, including “happenings, individuals present, weather conditions, physical/emotional actions, or reactions in others” (p. 680; Levine et al., 2002). The original event category, therefore, includes multiple distinct concepts, each thought to be represented by distinct neurobiological substrates (Caramazza and Shelton, 1998; Martin, 2007), so we elected to fractionate the event category into separately-scored detail types of activity, person, object, and miscellaneous (which included details ranging from weather descriptions to mentions of animals). This does not completely address the basic issue, first discussed by Levine et al. (2002), that any given category label is necessarily an approximation, but the current data nevertheless suggest that the current attempt at improving detail specificity was fruitful. One can consider the current modification of internal detail types to be a complement to recent work by Strikwerda-Brown et al. (2019) or Renoult et al. (2020), who similarly modified and expanded external detail categories when studying memory recall in patient populations; or work by Sheldon et al. (2019), who reorganized internal detail types into broad “perceptual” and “event-based” categories.

Some prior work has separately considered “construction” and “elaboration” phases of retrieval, with the former being associated with the development of a coherent scenario and assembly/reconstruction of relevant details and the latter being associated with the scenario then playing out in the mind's eye (Addis et al., 2007, 2009a; McCormick et al., 2015). Others have suggested that detail-related retrieval processes tend to occur transiently and early during mental time travel (Thakral et al., 2017, 2020; but see Bellana et al., 2017). Given that the speaking period for each trial in this experiment is analogous to the “elaboration” phase of earlier work, one might therefore have expected to see reactivation effects falling in “typical” default/core regions rather than distributed across the cortex. Instead, the current findings demonstrate that dynamic reactivation effects, once accounted for, are present in broadly distributed brain networks throughout prolonged periods of recall. That is, although an initial construction period is surely involved in retrieving a specific event, details continue to be accessed throughout the re-experiencing of an event. These observations therefore inform results previously reported by McCormick et al. (2015), who described widely distributed, primarily posterior cortical connectivity changes associated with elaboration phase during covert recall. Based on the current data, one might conclude that these effects were actually an amalgamation of the category-specific reactivation effects observed in the current data.

### Reactivation effects were grounded in category-selective cortex

Having identified dynamic, category-specific reactivation effects, the last and most important remaining question concerned their localization. Consistent with reinstatement effects predicted in prior laboratory experiments or computational models (Wheeler et al., 2000; Norman and O'Reilly, 2003; Vaz et al., 2019; Bone et al., 2020), and with the wide literature on conceptual knowledge more generally, reactivation effects associated with each examined detail category overlapped considerably with category-selective cortex as defined using a functional localizer task (Fig. 5). For place and object details, ∼30% of the localizer-defined cortex was subsumed by their respective reactivation maps, while the social categories exceeded 75% overlap with their corresponding localizer-defined mask. One might, at first, be concerned that the match between the social details and a task-negative comparison map is less obvious than the other condition pairings. However, the substantial overlap observed (both proportionally, and as an overall Dice similarity coefficient), would argue instead that the localizer-derived task-negative mask is a highly appropriate comparison point. Thus, as details are being recalled and described, cortical regions that support various forms of knowledge are dynamically reactivating and, we hypothesize, providing information to default/core regions to enable the rich experiences associated with mental time travel. Such a hypothesis would be consistent with prior reports that changes in event contents are decodable within regions of the default/core network during the production of (or when listening to) narrative descriptions (Simony et al., 2016; Baldassano et al., 2017, 2018; Chen et al., 2017b).

The relative differences in reactivation-related activity illustrated in Figure 4 further support the dynamic nature of detail retrieval. As one example, regions that support different aspects of spatial/scene processing are not required to contribute social information although they are involved in reinstating various Place-related details, whereas adjacent regions important for social cognition support reinstantiation of social details but do not particularly support reconstructing the space in which an event occurred (for related discussion, see Silson et al., 2019b). Thus, although the structures required to provide information are dynamically shifting throughout the recall of an event, a single network of regions can use the provided information to maintain a coherent internal event representation throughout a sustained period of recall (for related discussion, see Ritchey and Cooper, 2020). Future work specifically targeting dynamic network-level interactions in overt recall conditions could more directly test this hypothesis.

### What about the hippocampus?

It may seem surprising that the hippocampus, despite its strong association with autobiographical recall (Squire, 1992; Sekeres et al., 2018), did not feature more prominently in this report. However, hippocampal effects can be subtle, and while a whole-brain contrast of the autobiographical recall and picture description tasks did not identify significant hippocampal clusters, a targeted investigation of individual-specific hippocampal subregions using these same data identified posterior hippocampal differences between the two tasks (Gilmore et al., 2020), as one might expect based on prior literature. Thus, it seems reasonable to conclude that the hippocampus supports sustained periods of elaboration (see also McCormick et al., 2015; Chen et al., 2016) as well as initial event construction (Addis and Schacter, 2012). It should, however, be less surprising that hippocampal effects were not observed in later detail-specific reactivation analyses, as these always involved contrasting BOLD activity related to internal (episodic) details and thus the expected hippocampal activity would have been subtracted away.

### On the separation of semantic and episodic memory

In recent years, the relationship between episodic and semantic memory has become a focus of renewed interest in cognitive neuroscience (Renoult et al., 2012, 2019; Irish and Piguet, 2013). Of particular relevance here is increasingly fuzzy distinction of what constitutes an episodic or semantic contribution to retrieval (Renoult et al., 2019). In the present work, the reactivation effects of interest were grounded in nominally episodic details, that is, they related to a spatially and temporally specific occurrence (Tulving, 1972, 1983), but the regions associated with those reactivation effects respected “semantic” conceptual or categorial boundaries defined within the same cohort. Thus, data from the current experiment appear to resonate strongly with other emerging evidence that questions the clarity of the episodic-semantic distinction. Indeed, a complete separation may not be realizable. It may be, as Tulving (Tulving, 1984, 1985) argued some 35 years ago, that episodic memory is “embedded” within the semantic system. It would follow, then, that semantic (conceptual) knowledge is automatically accessed during vivid autobiographical recall.

Despite potential difficulties in completely separating episodic and semantic retrieval effects, future experiments based on paradigms similar to that described here appear to hold great promise. One could, for example, scan participants both while they view (encode) and later recall film clips to better understand the specificity of the effects observed in the current report (for related discussion, see Lee et al., 2020). Overt speech and verbal content scoring can also be applied to study cognitive phenomena beyond episodic memory. For example, one could also use a similar approach to study dyadic speech, perhaps under situations in which participants possess asymmetric knowledge of a topic. To the extent that semantic memory forms a fundamental backbone of cognition (Martin, 2016), it would seem that naturalistic, in-scanner speech should prove a versatile tool, indeed.

The current data provide clear evidence that the act of mental time travel not only requires regions commonly associated with autobiographical recall, but dynamically brings online distinct systems associated with the representation of scenes, objects, and people. These dynamics can be captured in real time, as the brain switches and back forth from one conceptual domain to another, with the use of overt, naturalistic recall.
